# Lean requirements traceability automation enabled by model-driven engineering

**DOI:** 10.7717/peerj-cs.817

**Published:** 2022-01-25

**Authors:** María-José Escalona, Nora Koch, Laura Garcia-Borgoñon

**Affiliations:** 1 University of Seville, Seville, Spain; 2 ITA Innova, Zaragoza, Spain

**Keywords:** Traceability, Model-driven engineering, Information fusion

## Abstract

**Background:**

The benefits of requirements traceability, such as improvements in software product and process quality, early testing, and software maintenance, are widely described in the literature. Requirements traceability is a critical, widely accepted practice. However, very often it is not applied for fear of the additional costs associated with manual efforts or the use of additional tools.

**Methods:**

This article presents a “low-cost” mechanism for automating requirements traceability based on the model-driven paradigm and formalized by a metamodel for the creation and monitoring of traces and an integration process for traceability management. This approach can also be useful for information fusion in industry insofar that it facilitates data traceability.

**Results:**

This article extends an existing model-driven development methodology to incorporate traceability as part of its development tool. The tool has been used successfully by several companies in real software development projects, helping developers to manage ongoing changes in functional requirements. One of those projects is cited as an example in the paper. The authors’ current work leads them to conclude that a model-driven engineering approach, traditionally used only for the automatic generation of code in a software development process, can also be used to successfully automate and integrate traceability management without additional costs. The systematic evaluation of traceability management in industrial projects constitutes a promising area for future work.

## Introduction

Traceability is defined by Drivalos-Matragkas et al. (2010) as the ability to chronologically interrelate uniquely identifiable entities in a way that matters. This very general definition pointing out the usefulness such interrelationships should have was later adapted by Lago, Muccini & van Vliet (2009) with reference to the life of software artifacts. CMMI (Team, 2010) defines bidirectional traceability as “an association among two or more logical entities that is discernable in either direction”. A more implementation-oriented definition is provided by the IEEE Standard Glossary of Software Engineering Terminology (1990), in which traceability is referred to as “the degree to which a relationship can be established between two or more products of the development process, especially products having a predecessor-successor or master-subordinate relationship to one another; for example, the degree to which the requirements and design of a given software component match”. This definition emphasizes the potential of traces in the requirements engineering domain, where traceability had its origin.

Both academia and industry recognize the importance of traceability and it is widely accepted that traceability can ease the maintenance of evolving software independently of the software development process employed (agile, waterfall, model-driven, *etc*.), particularly for large systems. Common software development company activities like managing changes in requirements, re-planning projects or validating whether requirements have been appropriately incorporated into software products all benefit from the efficient maintenance of requirement traces.

Traceability is strongly recommended in industrial standards like CMMI, which establishes a specific procedure (SP 1.4 Maintain Bidirectional Traceability of Requirements) in the Requirements Management Process Area at Maturity Level 2.

However, traceability implementation in industrial projects is limited for fear of the overheads it may involve. In practice, traceability often implies a qualitative improvement, but one which is usually difficult to measure. The difficulty of comparing software development with and without traceability management under almost identical conditions is also the main reason for the lack of systematic evaluations regarding traceability return on investment (ROI).

To address the high costs associated with trace maintenance and reconstruction throughout the entire life cycle of the software product from inception to deployment and subsequent maintenance, the present research focused on reducing the efforts required to achieve traceability, the ultimate aim being to obtain traceability almost “for free”.

The approach was to generate and maintain traces automatically during modeling phases, without the need for additional work by software engineers/developers. These automatically created traces are used to generate trace artefacts and provide information that can be passed from one phase to another one. This way, it is possible to trace how requirements are handled in the analysis stage and how they evolve in subsequent design stages. This approach is based on the Model-Driven Engineering (MDE) paradigm. In this regard, traceability is reduced to the management of traces between models used in the software development process; models that belong either to different levels of abstraction or, in some cases, to the same level (so-called vertical and horizontal traces). This paper describes how any methodology can be enriched using such an MDE-based approach to traceability—a procedure that has to be carried out just once by the methodology expert, or, exceptionally, more than once if the methodology is improved. This approach also requires a traceability support tool: *i.e*., a tool that can store trace links according to trace rules, monitor traces if source or target models are changed, and display warning and error messages in case of conflicts.

The application of such an approach to traceability management is, then, clearly a task that has to be defined and implemented by the methodology expert. Once integrated in the tool, it will be transparent to software developers, who will only see a monitoring mechanism for dealing with trace conflicts. A requirements traceability matrix can be used to manage traces between functional requirements and test cases, design specifications, and other artifacts.

The paper provides a theoretical foundation and describes how the approach was integrated into an existing methodology. The integration of trace creation, monitoring and maintenance into any existing model-driven software development methodology and its tool suite is illustrated using the Navigational Development Techniques (NDT) (Escalona & Aragón, 2008) for the development of web applications. Traces were created and monitored completely automatically. One pre-requisite for automation is tool support based on trace rules. These trace rules need to be defined only once for each methodology. From time to time, monitoring-based model maintenance may require decisions to be taken by the developer, but only if inconsistencies arise in the models.

The approach described was used in several industrial projects, including iMedea (G7 Innovation, 2021) in the healthcare sector, DILECO (2021) a European H2020 project in the aerospace sector, and SAGE (2021, https://investigacion.us.es/sisius/sis_proyecto.php?idproy=29761), focusing on electromobility in a Smart-City ecosystem. Part of the iMedea project is described in this paper to illustrate how the approach automates the generation of traces and supports their management.

The paper makes the following contributions:

1. A generic approach to MDE traceability that explicitly includes a tracing mechanism.
2. Extension of a methodology by trace and change management.
3. An example of its application in a real context.

The article is structured as follows: “Materials and Methods” provides an overview of the materials and methods involved in traceability concepts and the challenges they pose. It also looks at related works in the field of traceability, focusing on model-driven approaches with (semi-) automated tool support and their use in real industrial projects. “Results” presents the results obtained, which are a theoretical framework for requirements traceability based on the MDE paradigm and its integration into an already existing methodology, implemented and validated in its tool suite. The framework includes a metamodel and an extension of the development process. “Discussion” discusses the approach and “Conclusion” concludes with plans for improving it further.

## Materials and methods

### Traceability challenges

Since the 1990s, the advantages and problems of traceability have been discussed in several academic works, including the surveys of Gotel & Finkelstein (1994) and Winkler & Pilgrim (2010) and the articles of Haouam & Meslati (2016) and Charrada et al. (2011). The first, a cornerstone paper in requirements traceability research, identified the main problems of requirements specification. The second highlighted the gap between industrial needs for traceability integrated within the development process and existing academic proposals. Haouam & Meslati (2016) focused on the automated generation of traces but also on the updating and maintenance of (semi-)automated traces, while Charrada et al. (2011) proposed a benchmark for traceability covering artifacts typically produced in software system development processes and also end-to-end trace links. For further discussion of related works, the reader is referred to the next section.

**Figure 1:**
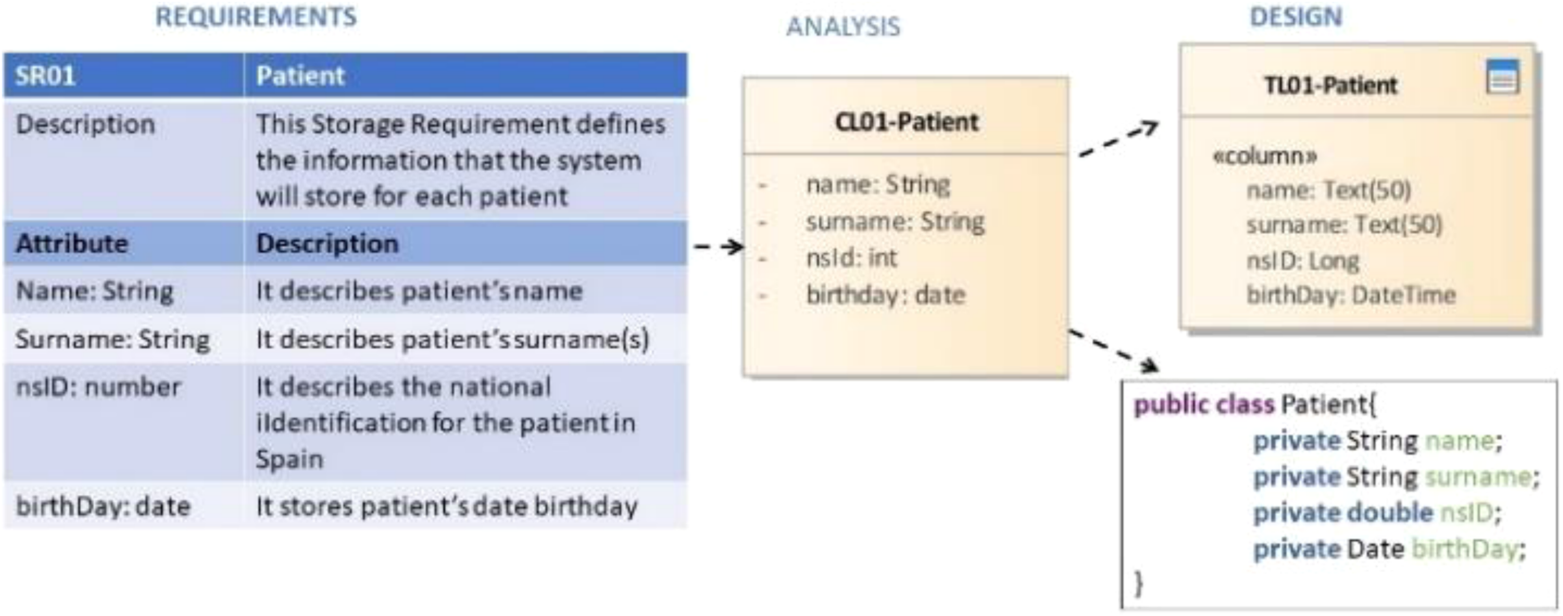
Excerpt of sample application. This image presents an example to illustrate the traceability of a storage requirement in the analysis and design phase.

**Figure 2:**
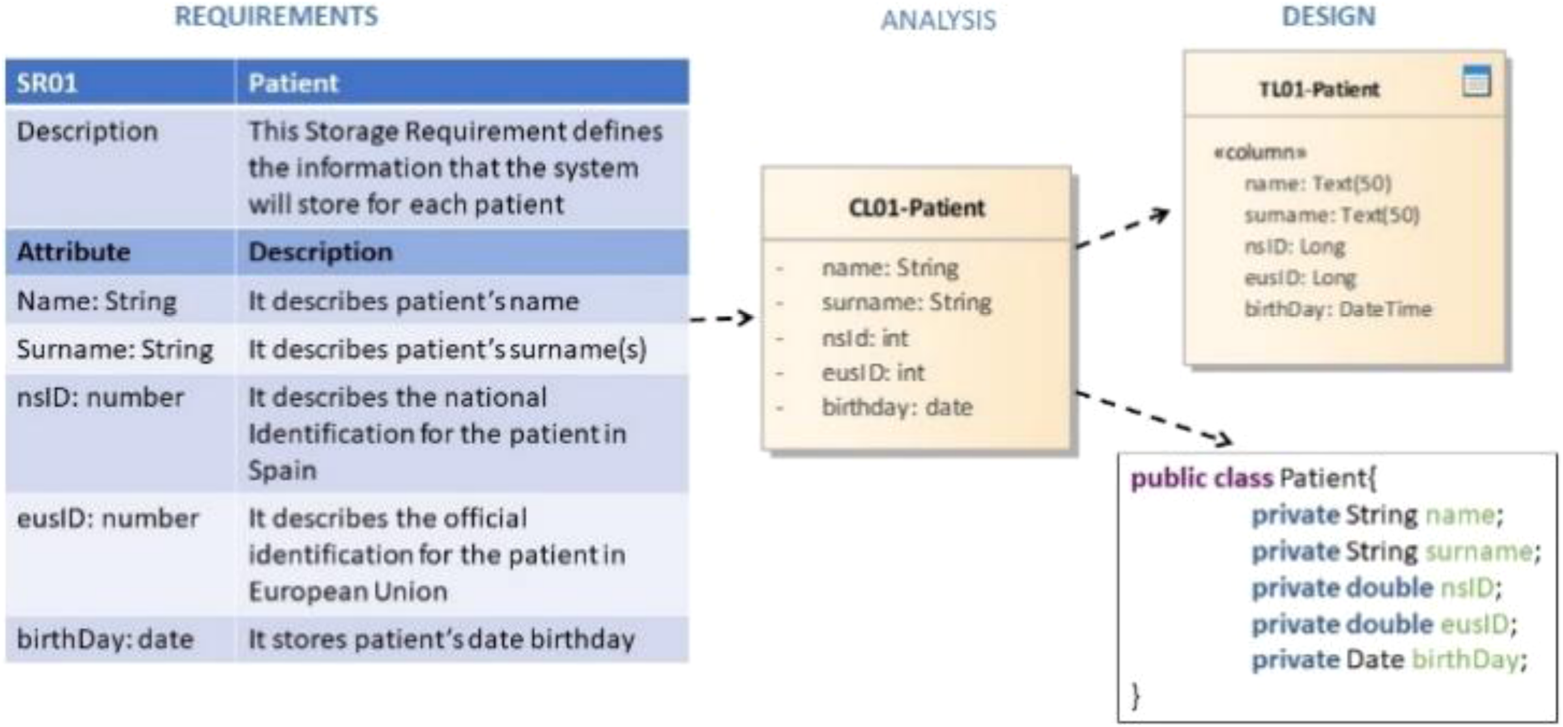
Changing requirements of the sample application (Excerpt). Example of traceability of Fig. 1 with a little change. It helps to illustrate the importance of the traceability management.

At this point, it would be useful to illustrate the importance of traceability with an example application from the iMedea project, a software solution for clinical history management in human reproduction environments. An excerpt of this application is shown in Figs. 1 and 2. A team of analysts is defining the requirements for a new medical system to be developed in a European country (for example, Spain). In this requirements phase, the requirements engineer and the health experts establish that for each patient the system has to store their name, surname, national health identification number (NSId) and birth date (as well as other information). This requirement could be modelled as a storage requirement or object, like the one shown in Fig. 1.

During analysis, such a requirement will be modelled as a class in which to store the patient’s information (identified in Fig. 1 as CL-01). In the design phase, the database designer also creates a table to store the corresponding information (TL-01). A programmer can later create, for instance, a Java class to support this class at code level (PatientJavaClass). There is evidently a connection between these four artifacts. In fact, each of them is derived from the one preceding it. Traceability means the capability of the software development tool to remember this kind of connection and use it to guarantee the coherence of the software artifact.

Going one step further, once the medical system has been initially deployed and implemented, changes in regulations make it necessary to store not only the national health identification number (NSId) but also the European health identification number (EUSId) for foreigners. The personal information requirement therefore changes, but so does the class at the analysis level, the table in the design level database and the code in the Java class, as can be seen in Fig. 2. If requirements are not traced, a simple change like this may imply the difficult task of analyzing which artifacts of the system are involved. A real software system has a high number of requirements, attributes, relationships, *etc*., so traceability is critical in order to guarantee consistency in software products.

Traceability is today also seen as a method for managing relationships between artifacts other than requirements (Winkler & Pilgrim, 2010): for example, for software maintenance, for evaluating the effect of a change or for calculating software costs. It makes it possible to assess the global impact of a change or a decision, *i.e*., to trace its effect. In an enterprise, traceability is a resource that helps to ensure software quality (Team, 2010).

Despite its advantages, however, traceability is not widely used in enterprises, or at least it is not considered a key factor (Cleland-Huang, 2006). Antoniol et al. (2017) analyzed why this is the case and came to the conclusion that traceability research should focus on the following challenges in order to make traceability useful in industrial projects:

- Ch.#1. Automatic generation of traces. In industrial projects, cost is a relevant factor. If traceability produces additional cost, it is difficult to incorporate into software methodologies. In enterprises, the generation and maintenance of a trace model should therefore be automatic, or at least automatic enough to guarantee no impact on project costs.
- Ch.#2. Tool support for trace model maintenance. This should (a) guarantee trace integrity in order to ensure that models are always updated, (b) offer mechanisms to easily identify and classify types of changes and their impact on the trace model, and (c) provide effective trace model management mechanisms that aid strategic decision-making regarding the pros and cons of introducing changes.
- Ch.#3. Return of investment (ROI) measurement. Indicators or metrics are required to measure ROI—a key factor in industry, and especially in the use of trace models.
- Ch.#4. Demonstration of the benefits of traceability in real examples, managed by real users. This requires close collaboration between academia and industry. In this regard, academia should avoid the use of non-real examples.

These challenges provided the inspiration for the present study. They concur with those identified by Tufail et al. (2017) in a survey which included ten challenges that could also be interpreted as traceability problems. This paper focuses on those challenges that are the most relevant to industrial applications, such as poor tool support, lack of guidance and commitment, and the different viewpoints of stakeholders. Challenges are further discussed in the section on related studies.

### Related work

The following research lines are relevant to the objectives of this study: (1) analysis of traceability challenges, (2) metamodeling traceability, (3) integration of automated trace management in model-driven engineering (MDE) tools, and (4) use of traceability in real software development projects. These lines cover similar aspects to those included in the characteristic schemata of the systematic mapping study conducted by Vale et al. (2017) for traceability in the area of software product lines. The present review opted not follow systematic guidelines and is not limited to one specific area of software. To the best of our knowledge, none of the currently existing approaches covers all these aspects or has a proven track record of providing a “low-cost” approach for automating MDE-enabled requirements traceability in several real software development projects.

#### A. Traceability challenges

The work presented by Gotel & Finkelstein (1994) is a cornerstone paper in requirements traceability research. Its authors describe the main problems of requirements traceability, including the lack of a common understanding, the need to allocate time and resources, and the gathering and maintenance of trace information.

Rempel & Mäder (2016) also focus on traceability difficulties, providing an assessment model and a comprehensive classification of possible traceability problems and assessment criteria for systematically detecting those problems.

A more recent literature review was carried out by Tufail et al. (2017). This review focused on requirements traceability techniques, models, and tools, offering detailed analysis and comparison and providing a set of comparative tables. It distinguished, among other things, between traceability metamodels, traceability process models and traceability information models. The work included a general list of 10 traceability challenges, and a more detailed analysis of traceability tools.

The present article focuses on the way its proposed approach could address traceability problems and challenges and overcome what are ostensibly the most relevant challenges identified in the above-mentioned papers, namely: the automatic generation of traces (Ch.#1), tool support for trace model maintenance (Ch.#2), return of investment (ROI) measurement (Ch.#3) and demonstration of the benefits of traceability in real examples managed by real users (Ch.#4).

The efficient management of traceability is another key factor to consider when combining data from different sources to generate more complete, improved and more accurate information. As such, it is also critical for information fusion. For information fusion to be successful, it is necessary to know the origin of the information in order to trace it in case of any change in the future. Trace management tools are therefore critical to guarantee the correct maintenance of fused information.

#### B. Metamodeling traceability

Several metamodels have been published during the last two decades covering different aspects of traceability, and in particular related to requirements traceability. As in the metamodel comparison of Carniel & Pegoraro (2018), in the present study a set of criteria were defined for comparing the metamodels relevant to the objectives in question. These criteria include support for: (1) textual, graphical and/or model representation of requirements, (2) associations between traceable elements, (3) horizontal and vertical traces, (4) definition of an algorithm or rules, (5) trace maintenance (6) change impact analysis.

**Figure 3:**
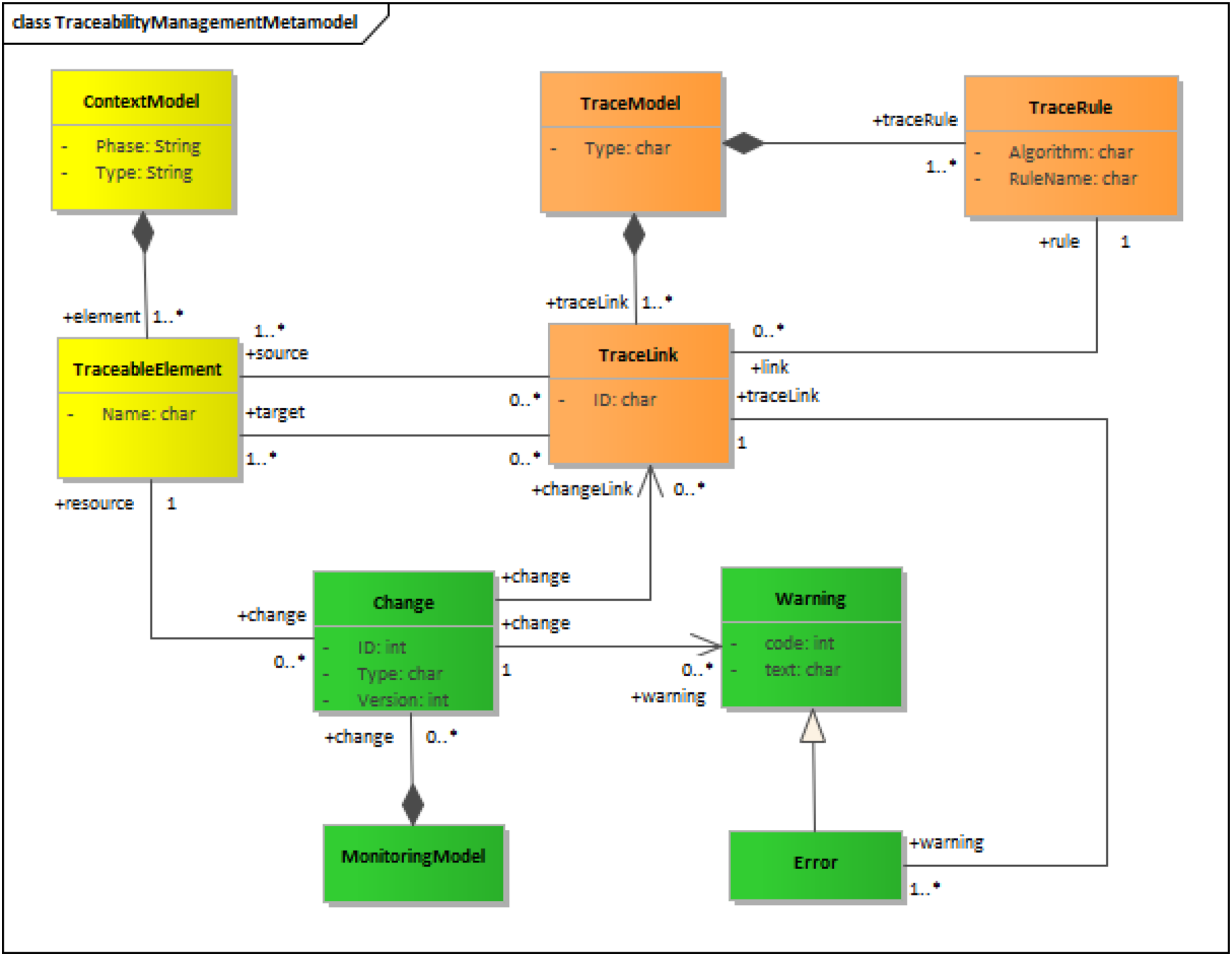
Traceability metamodel. This metamodel presents our approach for the traceability. It is divided in three sections: context model, trace model and monitoring model.

Most research and most metamodels in the field of traceability (*e.g*., Haouam & Meslati, 2016; Walderhaug, Hartvigsen & Stav, 2010; Sousa et al., 2008; Goknil, Kurtev & Van Den Berg, 2014) have to date focused on the creation of traces, *i.e*., on aspects (1), (2) and (3) above, but have neglected monitoring and maintenance, *i.e*., aspects (4), (5) and (6), which are the focus of the present work (see Fig. 3). The main approaches to traceability maintenance (Briand, Labiche & Yuea, 2009; Boronat, Carsí & Ramos, 2005; Cleland-Huang, 2006; Kassab, Ormandjieva & Daneva, 2009; Drivalos-Matragkas et al., 2010) are briefly discussed below.

Drivalos-Matragkas et al. (2010) graphically represent a tracing metamodeling language that covers tracing and maintenance concepts in a manner similar to that of the metamodel envisioned in the present study. Their approach, however, is state-based, unlike that of the present study, which is event-based and focuses on the detection of dangling links.

Briand, Labiche & Yuea (2009) focus on changes between two versions of a UML model (vertical traces only) and analyze the impact of those changes using formally defined impact analysis rules (written in Object Constraint Language). Traces between model elements are not included as first citizens in their metamodel.

Boronat, Carsí & Ramos (2005) had as their objective to provide generic traceability support to solve specific problems such as change propagation. Their metamodel provides a metaclass manipulation rule for each trace link, but change management is not included at metamodel level.

Cleland-Huang (2006) propose an event-based approach to traceability maintenance using observation to detect changes in the requirements models. These observes check then potential changes in the trace links. Potential changes in the trace links are then checked Unfortunately, this work provides no graphical representation of the approach.

Kassab, Ormandjieva & Daneva (2009) consider the tracing not only of functional requirements (FR) but also of non-functional requirements (NFR). Their metamodel includes metaclasses for both types of requirements and their associations, but metaclasses for change management are missing. Instead, they opt for XML-based representation and XQuery implementation for traceability management.

#### C. Integration of automated trace management in MDE tools

Traceability integration in a model-driven approach requires the definition or extension of metamodels and transformation rules for the automated generation and analysis of traces. The work involved in establishing these definitions is limited and required only once for each tool supporting a development methodology. The advantages of using a model-driven engineering approach are widely documented in the literature (Winkler & Pilgrim, 2010; Escalona et al., 2007; García-García et al., 2015). In particular, Winkler & Pilgrim (2010) provide an overview of research and practice in traceability and requirements engineering focusing on use of MDD for trace generation. The information presented is classified into four categories: basics, working with traces, practice, and solutions. The work addresses the generic use of traceability information, its visualization, and its usability, but does not address the exchange and analysis of such information.

Cleland-Huang (2006) focused on links between, on the one hand, textual documents and models and, on the other, documents and code. Walderhaug, Hartvigsen & Stav (2010) instead propose a generic MDD traceability solution based on guidelines and templates. The links proposed by Cleland-Huang (2006) are generated automatically but require acceptance or rejection by the users of the proposed tools. The main issues are the amount, granularity and quality of the links generated, so the authors discuss a set of metrics for evaluating the effectiveness of automated traceability. Although the amount of trace links generated in their approach is limited, the problem remains of how to maintain a list of links that may have become outdated due to the modification of artifacts.

In contrast to these approaches, Briand, Labiche & Yuea (2009) present a horizontal and vertical traceability impact analysis based on metamodeling and OCL constraints. Their metamodel includes tracing and monitoring concepts similar to those in the present study, but is dependent on the type of representation selected for the concepts (UMLDiagram and ClassDiagram, for example). However, this proposal focuses exclusively on change impact analysis, ignoring other traceability-related practices.

As part of the AMPLE project, Anquetil et al. (2010) presented a traceability metamodel focusing on trace links, trace context and model artifact types, while Goknil, Kurtev & Van Den Berg (2014) presented a metamodel that relates requirements and architecture models through traces (the approach proposed in the present study is even more generalizable than this, as it has no restrictions regarding model types). Traces are generated automatically but in an iterative process. The associated tool, which supports generation and validation, is based on model transformations in ATL and term rewriting logic in Maude. Another formal approach based on Maude is that adopted in a MOMENT project that uses QVT for model transformations and OCL constraints (Boronat, Carsí & Ramos, 2005) for the definition of generic operators. Haouam & Meslati (2016) describe a traceability maintenance approach based on model comparison, change detection, classification, and link evolution. Here, traces are generated automatically and the updating process is (semi)-automatic.

#### D. Use of traceability in real software development projects

Reports on the use of traceability in real software development projects are difficult to find. Many approaches are illustrated using textbook examples, as is the case of Walderhaug, Hartvigsen & Stav (2010) and Briand, Labiche & Yuea (2009). In contrast, the approach proposed in the present study has been used in several real software development projects. A systematic evaluation is not included in this paper as it is still work in progress.

The present approach also differs from that of Walderhaug, Hartvigsen & Stav (2010), who focused on a conceptual solution for tool integration and implemented a set of plug-ins for the Eclipse platform using Rational Software Architecture (RSA), insofar that its goal was to facilitate the extension of development tools without using complementary tools.

The survey by Winkler & Pilgrim (2010) focuses on traceability in the areas of both MDE and requirements engineering. The authors mention the gap between industry and the solutions proposed by academic researchers, and highlight the need to support traceability as part of development processes.

One interesting work on best practices for the establishment of automated traceability in enterprises is that presented by Cleland-Huang (2006), in which the following criteria were established to make automated traceability possible in industry: the establishment of an appropriate environment, the creation of traceable documents, and automated trace processes. The second criteria is not relevant for an MDE approach.

Haouam & Meslati (2016) validate their work with a study focusing on two research issues: the reduction of the manual effort required to generate and maintain traces, and maintenance quality in comparison with manually maintained traces.

## Results

### Supporting traceability

Model-Driven Engineering techniques are mainly used for the automatic generation of code in the software development process (Hutchinson et al., 2011). MDE also plays an important role in other software engineering areas such as software testing, supporting the generation of test cases. The aim in the present study was to demonstrate the potential of MDE in terms of traceability, since it allows traces to be recorded as a by-product of model transformations.

#### A. Building upon an MDE approach

The Model-Driven Engineering paradigm consists of raising the abstraction level of software development. It is based on models, transformations, and appropriate modeling and transformation languages. Models are the key artifacts during definition, design, implementation, and deployment. There are two kinds of modeling: metamodeling and design level modeling, corresponding to two different levels of abstraction and providing, respectively, abstract and concrete syntaxes.

At first glance, this would appear to introduce a high degree of complexity, but that is not the case. Source and target metamodels define the relationship between concepts and need to be modelled only once. In the same way, model transformations are only defined once, unless the metamodel changes.

At design level, target models are generated automatically from source models, which are constructed on the underlying source metamodel. This means that additional modeling efforts are not needed, no matter how many models the transformations are applied to. Following the same principles, traces can be recorded during the transformation-based process which generates the target models. A set of traces constitutes a trace model, which can be generated automatically once the tracing rules are established: *i.e*., once the relationships between software artifacts of the same or different levels of abstraction are known.

**Figure 4:**
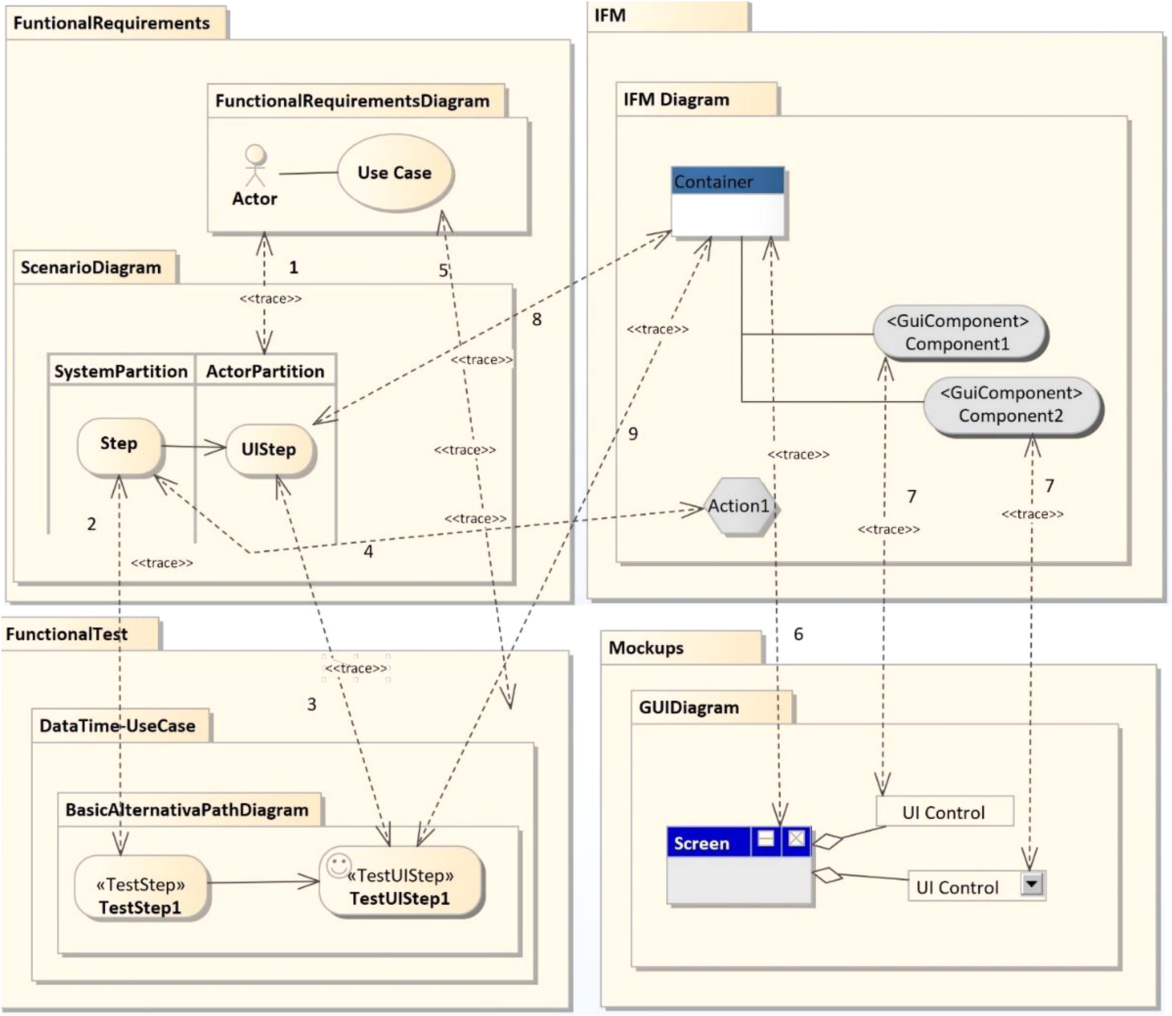
Excerpt of relationships between model elements. This model presents an example of our metamodel instanciation. It includes requirements models, concretelly: functional requirements, IFM, Functional tests and mockups.

The present study focused on bidirectional traces, traces going both backwards and forwards: *i.e*., from the origins through development and specification to subsequent deployment and use, and back to the initial requirements. It also distinguished between vertical, horizontal and evolution traces. Vertical and horizontal traceability refers to relationships between software artifacts at different or the same levels of abstraction and is also known as intra and inter traceability. Evolution traces are links that indicate different versions of the same software artifact. Examples of traces are shown in Fig. 4.

To implement an MDE approach (Brambilla, Cabot & Wimmer, 2012; OMG, 2021, https://www.omg.org), metamodels are represented using different languages, such as the Unified Modeling Language (UML) and Domain Specific Language (DSL). The languages most frequently used to specify transformations are Query-View-Transformation (QVT) and the Atlas Transformation Language (ATL). Modeling and metamodeling activities are supported by frameworks like Eclipse or UML CASE tools like Enterprise Architect or MagicDraw, while environments like SmartQVT and EMF (Eclipse Modeling Framework) for ATL can be chosen to support the transformations and the tracing process.

There are different ways to represent traces. One of the most commonly used techniques is the traceability matrix (TM). More challenging issues are how to maintain consistency in the TMs required for the target models in case of changes in the source models and the implications that changes in the target models may have for the source models. It is always possible to completely regenerate target models and TMs, but this solution is neither economic nor possible if the target models have been adjusted.

The following sections describe the proposed MDE approach. It is an approach that goes beyond the automatic generation of target software to actually document all traces resulting from the generation process, and manage those traces in order to discover and repair inconsistencies and identify conflicts when source or target software models are modified.

The approach is described at three different levels of abstraction: (1) a general theoretical level, independent of specific software development techniques and technologies, (2) a practical, methodological, level that is tool-dependent but project-independent, and (3) a project-related example.

For the theoretical level, a metamodel was developed which describes relationships in an MDE-based approach to traceability management (see Section B below). The practical and project-related levels are presented later, in the *Tool Support for Model-Driven Traceability* section.

#### B. Metamodel for traceability

Traceability management comprises the creation and maintenance of tracing models. Maintenance refers to changes in the models of the different software development phases. A (semi)formal specification of this traceability management approach was obtained using metamodeling as the description technique.

The proposed metamodel is similar to several existing metamodels mentioned in the *Related Work* section. However, it differs in its explicit metamodeling of the traceability mechanism and the change management elements. These are represented by the metaclasses TraceRules, Change, Warning and Error (see Fig. 3).

The key aspects in traceability management are identification of products from the specific contexts to be traced and the creation and monitoring of the traces detected. These aspects are represented in the metamodel shown in Fig. 3 as context, tracing, and monitoring models, represented in turn by the UML classes ContextModel, TraceModel and MonitoringModel.

Although colors have no semantic value in UML models, they are used in Fig. 3 to visualize the different aspects addressed by the metamodel: the context— that is to say, the models of the software development process (requirements, design, testing…)—is depicted in yellow, the traces in orange, and the monitoring of the models’ evolution in green.

The trace model is modelled as a composite of trace links relating elements of a source context model (for example, a requirement use case model) with the elements of a target context model (for example, a class model in the design phase). The related elements in the context model are the so-called “traceable” elements in the metamodel. These concepts are represented in the metamodel by the classes TraceableElement and TraceLink.

The relationship between source and target elements is based on predefined trace rules, which are explicitly metamodeled by the class TraceRule. Although denominated rules, they may comprise any type of algorithms for the creation or checking of traces.

TraceableElement describes any artifact in a context model and is identified by its attribute name. In the example of the patient (Fig. 1), for instance, a TraceableElement might be the storage requirement (SR-01), the class (CL-01) or the database table (TL-01), but also their attributes.

TraceLink and TraceRule are characterized by an ID and the definition of an algorithm. Every TraceLink has at least one source and one target TraceElement. TraceLink(s) for a software system are generated based on the TraceRule(s) definition for a specific methodology. A trace rule provides a formal description of the relationship between different elements of metamodels. In the example of the patient, a TraceLink would therefore be the relationship between the Patient Storage Requirement (SR-01) and the Patient Class in the analysis model (CL-01).

The proposed metamodel supports bidirectional TraceLink(s). Source and Target represent the directions in which transformations are executed to create the links. Vertical and horizontal links are both supported, depending on whether Source and Target belong to different versions of the same model or to different models.

Each context model consists of a set of traceable elements, while a tracing model is the set of trace links. ContextModel is therefore specified as a composite of TraceableElement(s), and TracingModel as a composite of TraceLink(s) and TraceRule(s).

The proposed metamodel includes explicit change management, indicating the impact changes have on the models. Context models are therefore monitored to detect changes in their traceable elements. Changes imply the need for traceability maintenance 0. Each modification of an element in a source context model is analyzed and may result in the automatic modification of the target context models and, if possible, of the corresponding trace links. Similarly, changes in a target context model may have implications for the source models. In both cases, a warning message alerts the user about the changes. If a conflict cannot be resolved, *i.e*., adjustments cannot be uniquely identified, an error message will be generated since a user decision is required.

In the example cited previously, the inclusion of the new European Health Identification Number attribute in SR-01 causes a change in both the class and the table because it requires a new attribute to be added to each of them.

The concepts described above regarding the monitoring and evolution of models are included in the traceability metamodel as a set of metaclasses and relationships. Unlike existing traceability metamodels, the proposed metamodel explicitly models the impact of changes, including those in the MonitoringModel, Change, Warning and Error classes. An error indicates that the associated trace link is no longer valid and constitutes a special instance of warning, so the relationship is specified as an inheritance.

Just as the ContextModel is specified as a composite of TraceableElement(s) and the TracingModel is specified as a composite of TraceLink(s) and TraceRule(s), the MonitoringModel is defined as a composite of Change(s).

#### C. Applying the MDE-based traceability approach

The traceability metamodel presented in the previous section is what is known in MDE terminology as a platform-independent model (PIM); that is to say, it is independent of the technology selected to develop the software. Even more importantly, it is also independent of the methodology used for the software development. This means that any model-driven software modeling methodology can implement traceability, instantiating our traceability metamodel and implementing the automated generation and monitoring of traces in the tool that supports the corresponding methodology.

This section describes a process for integrating traceability management in an existing methodology M with tool support T. In this process, it is important to distinguish the following roles:

- Methodology Expert is the person who manages or defines a concrete methodology M and who will implement the extension of M in the corresponding tool in order to support traceability.
- Developer is the person or team who uses methodology M and tool T to define their software projects.

**Figure 5:**
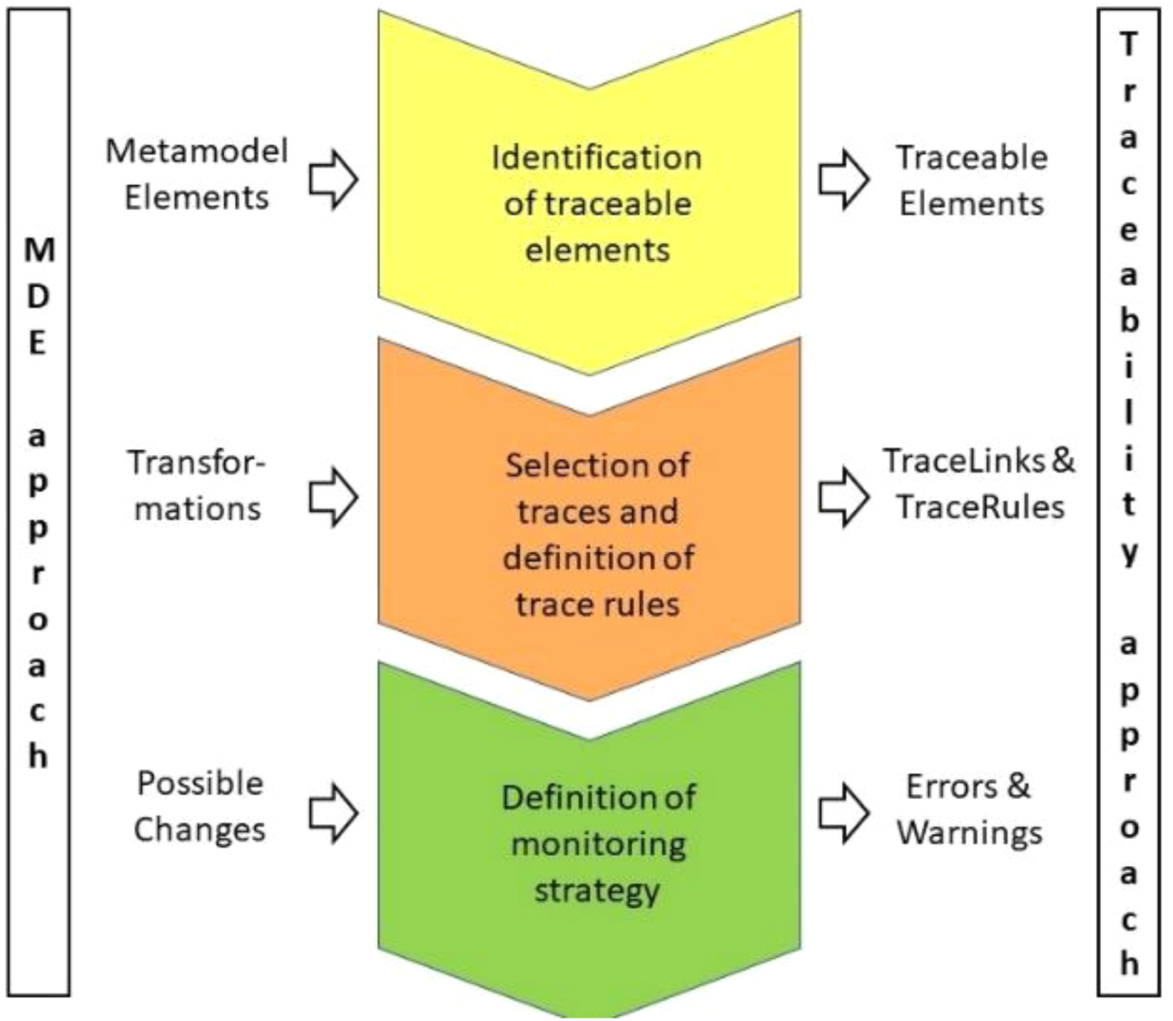
Process for applying our MDE-based traceability approach. This figure presents the proceedure that a developer has to follow to apply our approach.

The process of enriching methodology M with the proposed MDE-based traceability approach is mainly an activity that has to be performed just once by the methodology expert, or, exceptionally, more than once in the case of improvements to M. It consists of the following steps. For a graphical overview, see Fig. 5.

1. Selection of the subset of artifacts in methodology M that tool T has to trace, and storage of the corresponding information in, for example, a traceability matrix. These selected artifacts are instances of Traceable Elements (see Fig. 3).
2. Identification of the traces, based on the transformations observed between the artifacts selected in Step 1. Using these TraceLinks, the methodology expert has to define the TraceRules. These rules establish which artifact A is traced with which other artifact B, and its conditions, so that changing A necessarily implies modifying B to maintain information consistency. These rules are in general transparent for the developer, who uses the methodology M support tool to implement projects but has no knowledge of the rules that are used to generate the traces.
3. For monitoring, the process needs to identify the changes in TraceableElement(s) in the models, and the consequences of those changes. It must then decide if an automated solution of conflicts is possible: *i.e*., if a warning or error message should be communicated to the developer. This is an event-based approach to traceability maintenance.
4. The methodology M support tool then has to enable the management of Warning and Error messages.
5. The methodology expert has to define the way the MonitoringModel will be displayed to developers, by selecting, for example, a traceability matrix.

To summarize, a traceability support tool has to be able to (1) store trace links according to the trace rules in an appropriate format, *e.g*., a traceability matrix, (2) monitor the traces if source or target models are changed, and (3) display warning and error messages when conflicts are detected.

It can thus be concluded that the application of the proposed traceability management approach is a task that has to be defined and implemented by the methodology expert. Once integrated in the tool, it will be transparent to software developers, who will only see a monitoring mechanism for dealing with trace conflicts.

### Tool support for model-driven traceability

In this section, the proposed traceability management method is validated using Navigation Development Techniques (NDT). As mentioned in “Applying the MDE-Based Traceability Approach”, however, it can also be applied in other model-driven approaches like UML-based Web Engineering (UWE) (Koch & Kozuruba, 2012), MockupDD (Rivero & Rossi, 2013) and Web Modeling Language (WebML) (Brambilla & Fraternali, 2014).

The process presented in the previous section will be applied to integrate our approach in the NDT tool suite. The approach could be integrated in the same way in other software development tools, particularly in web application development tools. This section also illustrates its application in one of the real projects that were developed using the approach.

#### A. Brief overview

NDT is an MDE methodology that mainly focuses on web system requirements and analysis. Well-accepted in the industry (Escalona et al., 2007), its tool is implemented as an Enterprise Arquitect plug-in (García-García et al., 2015) and covers all phases of the software development life cycle. The latest version of NDT was improved with Design Thinking principles such as user empathy, early user incorporation, and the generation of prototypes. It supports three main phases in the software development lifecycle: conception, definition/design, and operation. Each of these phases is divided into a set of activities, such as functional requirements, capture, or architecture definition.

In this paper, NDT is used to show how the proposed approach allows for an extension of MDE-based software development methodologies integrating traceability support. The paper also shows how this traceability approach can then be used in enterprise projects. It does not aim to present a complete description of the NDT lifecycle. In this case, priority was given to the definition/design phase, focusing on three activities: design, construction, and verification and validation. The example shown corresponds to design activity.

The team’s main tasks during this activity were to define, analyze and design the following set of models for the future system:

- Functional Requirements, represented as a set of use cases that can be enriched with activity diagrams or scenarios.
- Conceptual Models, for the static view of the system.
- Mockups, to represent the screens of the system to be built. These were derived by transformations from prototypes and provide trace links between design and software conception.
- Non-Functional Requirements, used to document aspects of the system like usability, reliability, and security.
- Functional Testing, which was automatically derived from the functional requirements by transformations and offered a first validation of the requirements.
- Interaction Flow Model (IFM), which provided the relationships with all the other models and was the basis from which the architecture model was derived.
- Architecture Model, which made it possible to represent the software architecture—that is to say, the relationships between software components coded in different programming languages—and, more specifically, to model the model-view-controller pattern. Micro services and event-driven patterns will be considered in future versions.

#### B. Extending the methodology

The NDT models were built using the elements provided by the corresponding metamodels of the methodology, a so-called Domain Specific Language (DSL). Just as elements of the metamodels are related, so are those of the NDT model. These relationships are the basis for defining transformations and trace rules. The development team is able to see that an artifact is connected to others of the same system, but cannot see the metamodels.

In the first phase, prototypes are defined and, from these prototypes use cases can be generated. From the use cases, the methodology allows functional test cases to be generated. If the team detects an error or a problem in a functional test case, they can trace it back and find which user(s) validated the prototype in the Software Conception phase. In this regard, trace generation is automatic and trace management is semiautomatic, since the team needs to intervene to find a solution for any traceability problems that are detected.

Models of each phase are connected to other models of the same phase and to models of other phases—these are the horizontal and vertical traces mentioned earlier. These rules have now been specifically established and hardcoded within the tool. The traces are depicted in Fig. 4 which, to aid readability, shows only a representative excerpt of models corresponding to the Software Definition phase and a limited number of relationships. Figure 4 includes Functional Requirements, Mockups, Functional Testing and IFM models. A relationship between two elements in the figure means that there is dependency between those two elements. One of them is the source and the other is the target of the trace. For instance, the relationship marked with a number 5 means that the use cases are directly related to functional tests, and that a TraceLink (see Fig. 4) should exist between them.

In Fig. 4, only vertical traces are included, as the example used only relationship types 2, 3 and 5, but the NDT suite also supports other kinds of traces, like horizontal and evolution traces. These relationships were used to implement the design basis for the ContextModel and the TraceModel (see Fig. 3).

**Figure 6:**
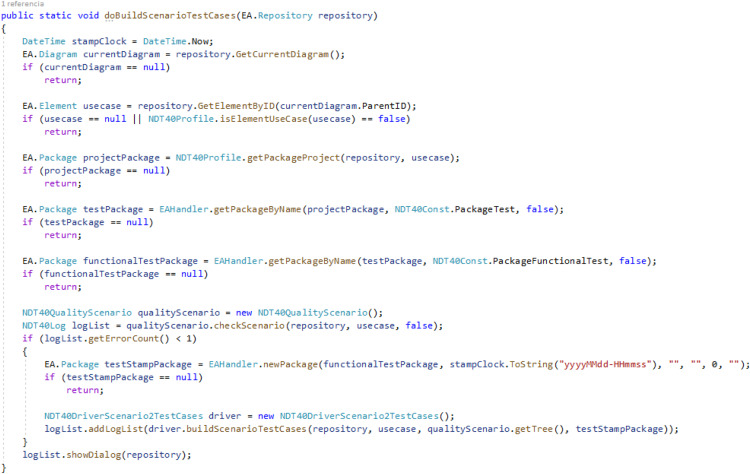
A specific trace coded in NDT-Suite. This image presents a part of code to illustrate how NDT-Suite implements a concrete trace.

Figure 6 shows a specific trace coded in the NDT suite. Specifically, it depicts the trace that was established between Step and UIStep from ScenarioDiagram, and TestStep and TestUIStep from FunctionalTest.

#### C. Example

To visualize the use of the proposed traceability metamodel, this section presents one of the real industrial software projects in which this traceability approach was applied. The objective of the iMedea project (G7 Innovation, 2021) is to create a software solution for clinical history management in human reproduction environments. It is a highly complex system that supports all functional requirements for the study and treatment of reproductive problems and diseases, and also non-functional requirements such as a complex data security system, support for medical standards and 24*7*365 maintenance mechanisms.

The system includes more than 75 different use cases, including *in vitro* fertilizations, human sperm donation and human artificial insemination processes.

**Figure 7:**
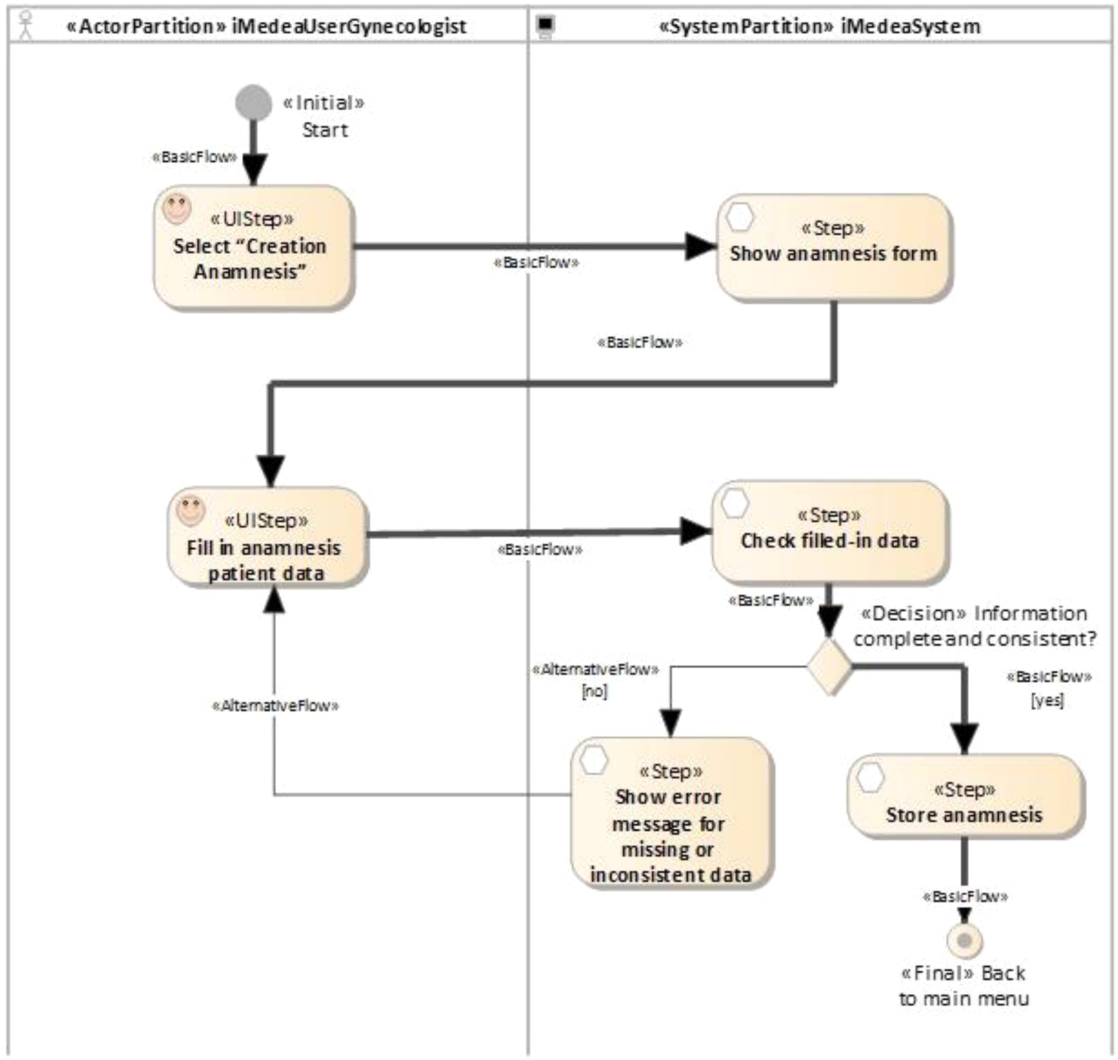
iMedea functional requirement: anamnesis creation. This figure presents an example of a function requirement in the iMedea project. Concretelly, it illustrates the anamnesis creation.

Here, the proposed traceability management approach is illustrated in the functional requirement “Anamnesis creation”, although the same approach was used for all the system’s requirements. The first time a patient attends a clinic, a set of standardized data corresponding to that patient’s clinical history is collected. That data set is called the anamnesis, or medical history. Figure 7 shows an activity diagram illustrating a simplified version of this use case. It represents the main flow (depicted with a thick line) and an alternative flow (the thin line). Whenever a new patient is registered, a gynecologist (iMedea user) will perform the anamnesis as a first step in collecting a large amount of data from the patient.

When the user selects the option “Anamnesis” the system displays the corresponding form. The user inputs the required information and the system checks that the data is correct and complete. If it is, the system creates the anamnesis and returns to the starting point. If not, an error message is generated and the system returns to the anamnesis form.

#### D. How traceability is implemented

The methodology outlined in Section A is fully integrated into the NDT suite, a set of tools that supports development teams in the application of MDE technology and traceability. The tool and the example described above in Section C were used to validate the proposed approach.

The tools required for implementing traceability were:

- **NDT-Profile**. As explained in the previous section, concepts used for each type of model in the methodology are modelled as metaclasses. A set of metamodels was defined for these related concepts. The metamodels, too, were interrelated. For each metamodel, a specific UML Profile was created which defined a DSL (Gutiérrez, Escalona & Mejías, 2011) that could be used in practice to model without requiring familiarity with metamodels. Actually, NDT offers a set of tools that can be used to draw different models, including prototypes, functional requirements, and test cases, while ensuring that the metamodels are correctly instantiated. This tool made it possible to implement the DSLs as an Enterprise Architect plugin. Relating this tool with the metamodel of the proposed traceability approach (see Fig. 3), it was also possible to build the ContextModel and its TraceableElements (the yellow area in Fig. 3).
- **NDT-Driver.** This tool supports the MDE generation of models: *i.e*., the obtaining of target elements from source elements. It implements the transformations defined in the methodology in C# language. The creation of the TracingModel (the orange area in Fig. 3) with the corresponding TraceLink(s) is a completely automatic activity. Whenever a transformation is executed, this tool stores the source, the target, and their relationship in an internal data base so that the relationship can be updated to reflect future changes or product evolution.
- **NDT-Quality.** This tool permits the automatic checking of models. It is the tool in charge of the MonitoringModel identifying the changes (the green area in Fig. 3). It deals with each error by sending a warning if a change is detected, or an error message to the team if a problem is detected and cannot be solved automatically. Conflicts are thus resolved semi–automatically, while conflict detection is automatic.

**Figure 8:**
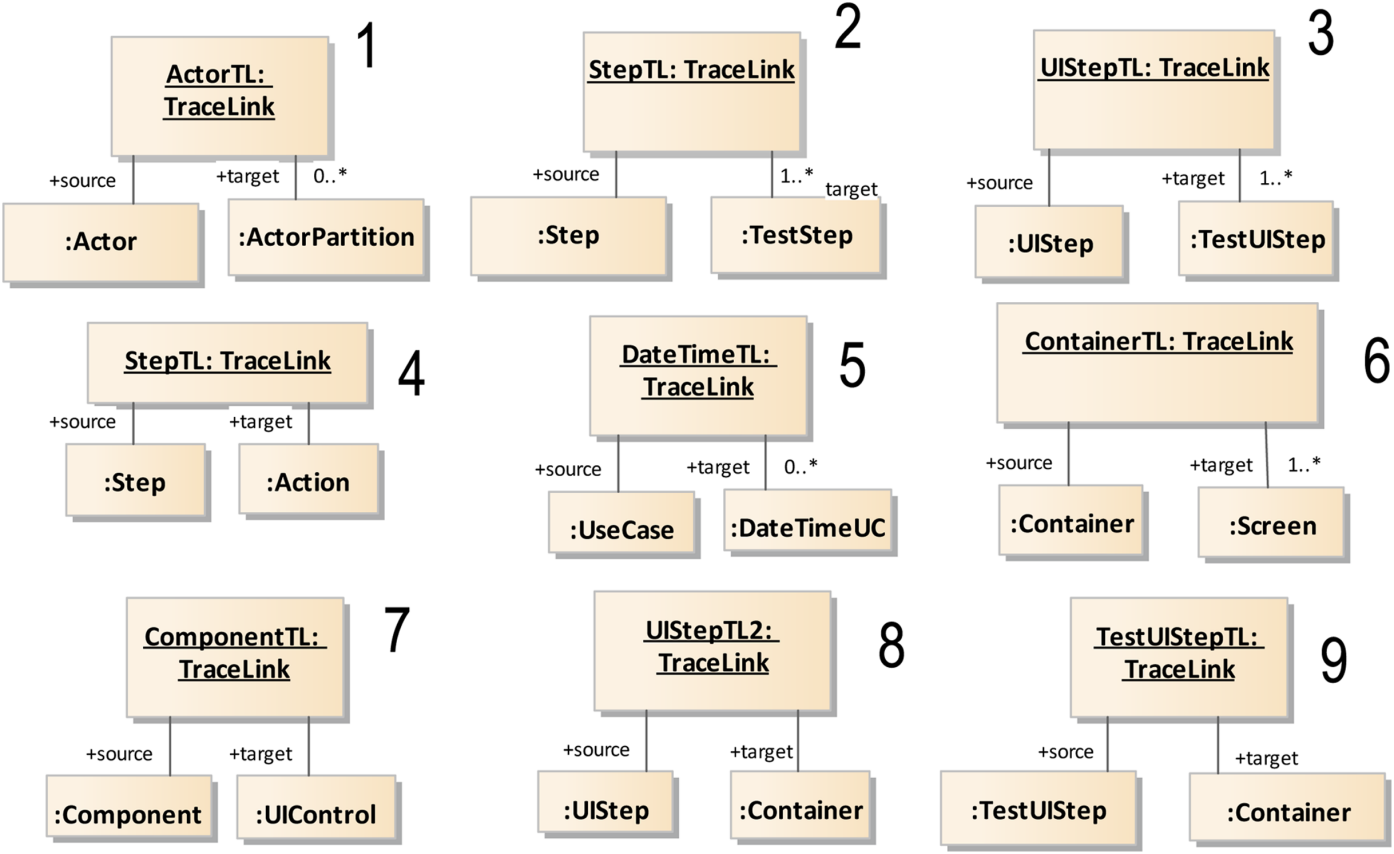
Instantiation of the traceability metamodel. The images illustrate an object class to represent how to instanciate the metamodel. An asterisk (*) represents multiplicity according to UML.

**Figure 9:**
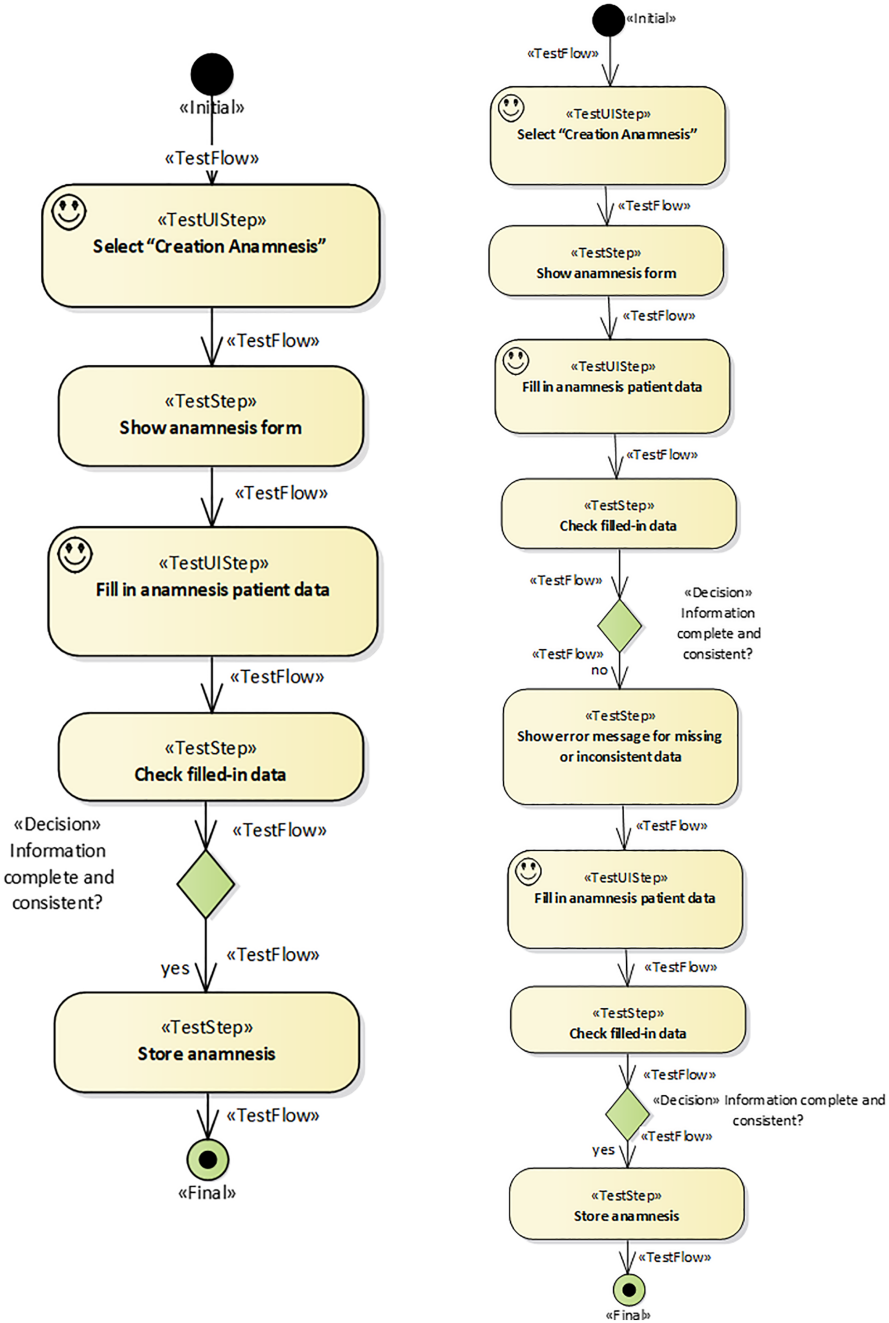
Excerpt of TestCases generated automatically. This figure present how the test case can be generated from requirements using transformations in our example.

Traceability is implemented in this tool environment by automatically generated bidirectional relationships. For this purpose, a set of trace rules was used which followed the schema provided by the traceability metamodel presented in Fig. 3. Figure 8 shows an UML object diagram with nine different trace rules, which in turn represent the relationships in the UML diagram between the elements of the different design models shown in Fig. 4. Each number in Fig. 8 refers to a relationship in Fig. 4. The example shown in Fig. 9 explains how relationships were automatically generated. One of the transformations generated functional tests from the activity diagrams associated with use cases (see the relationships numbered 2, 3 and 5 in Fig. 8). For each path in the activity diagram, a scenario was generated corresponding to a test case. The diagram in Fig. 7 shows a main path and an alternative path, conditioned by a loop. The tool could be configured to guarantee the desired degree of coverage. In this case, it was configured to consider only one loop and to generate only two test cases. The tests generated automatically were those corresponding to the diagrams depicted in Fig. 9.

#### E. Automated generation of traces

The objective of this work was not to describe in detail the method of generating tests but to analyze how relationships are created. For more details on the process of systematic evidence generation, the reader is referred to Gutiérrez, Escalona & Mejías (2011). At least one TestUIStep is generated from each UIStep and at least one TestStep is generated from each Step. In this case, as the loop was not considered, only one element was generated for each UIStep and for each Step. In the generation process, a scenario, represented as a simple activity diagram, shows the steps that a user should execute in order to partially validate a part of the activity diagram represented in Fig. 7. In the previous scenarios, decisions and values were predefined by the transformation that generates each test. Thus, although the decision element is retained for the sake of readability, only one path is given for each test.

**Figure 10:**
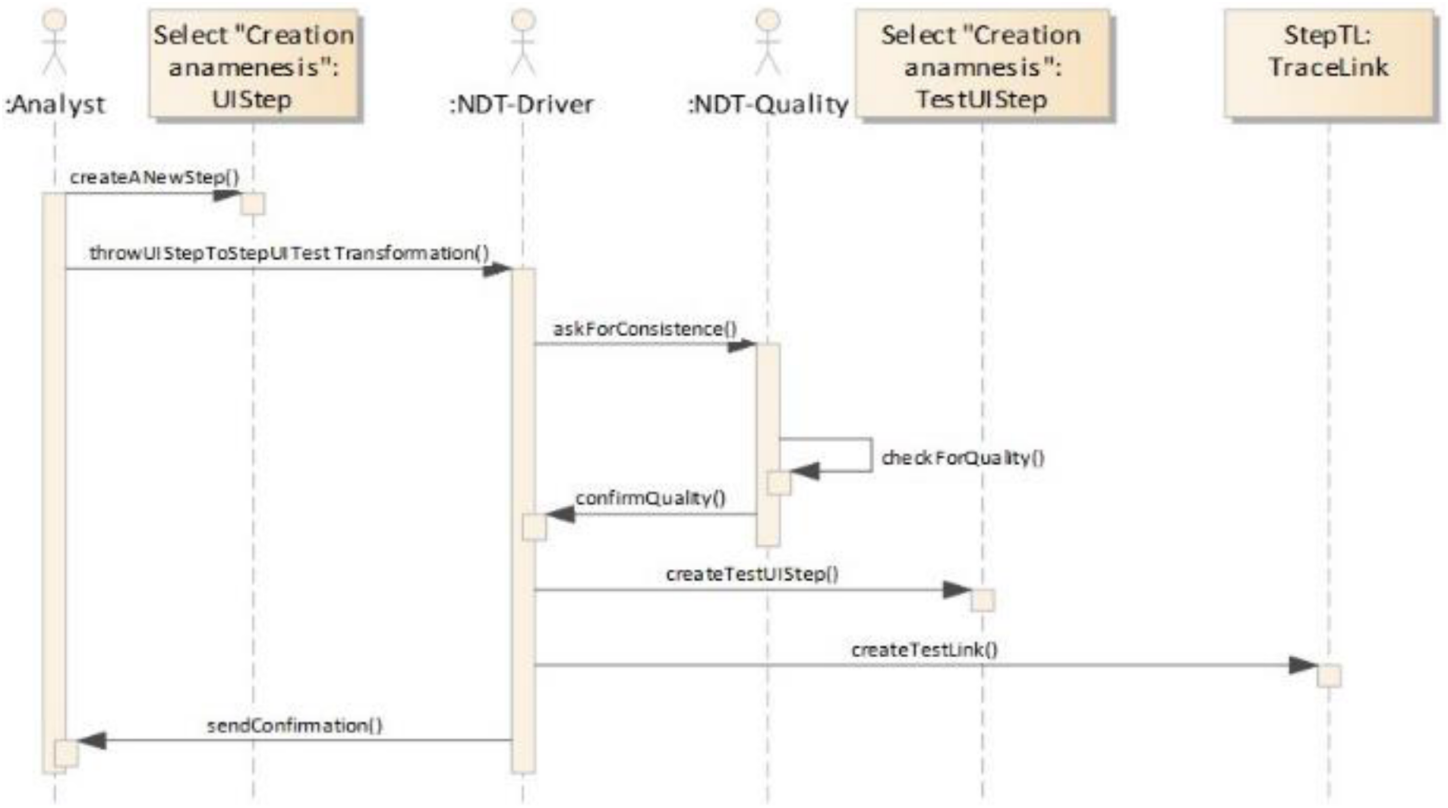
Building the TracingModel. This figure presents a sequence model that describes the process to generate the tracing model.

With this in mind, the following sequence diagram shows how the tools interact to generate these relationships (see Fig. 10).

When the developer creates the UIStep “Creation Anamnesis” and executes the transformation to generate test cases, the plug-in NDT-Driver asks NDT-Quality if the corresponding activity diagram is consistent and conforms to the relevant methodological principles. If it is OK, the TestUIStep “Creation Anamnesis” is created, together with a TraceLink object that stores this relationship (denominated StepTL in Fig. 7). The developer is notified of the correctness. The same process is performed several times, depending on the loops and decisions (in this example, only twice) for each activity in the activity diagram.

**Figure 11:**
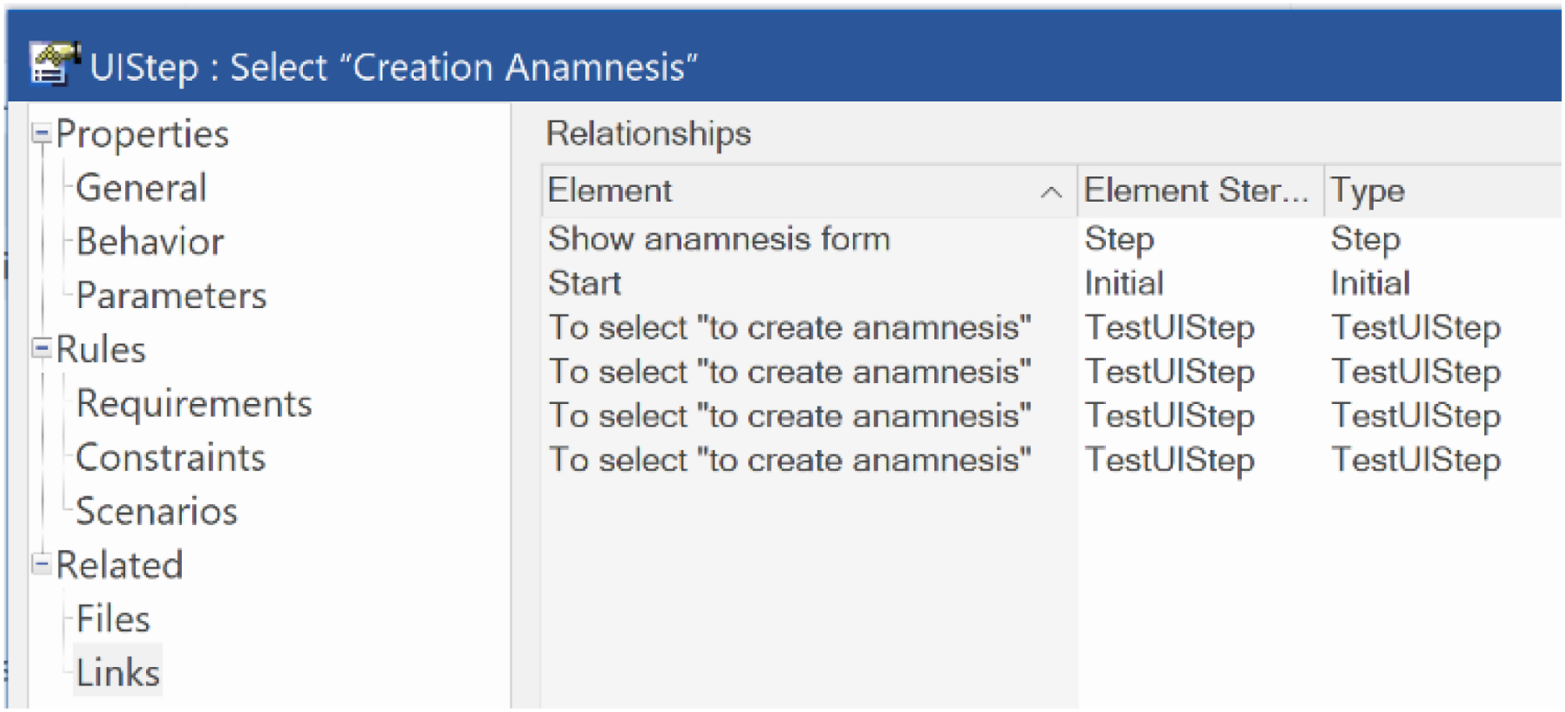
Elements related to the “Creation Anamnesis” activity. This screen shows how the tracing model was generated in the tool.

TraceLinks can be visualized in two different ways. Firstly, clicking on an element traces all elements related to that element. In this example, Fig. 11 shows the trace elements when the “Creation Anamnesis” activity is selected.

**Figure 12:**
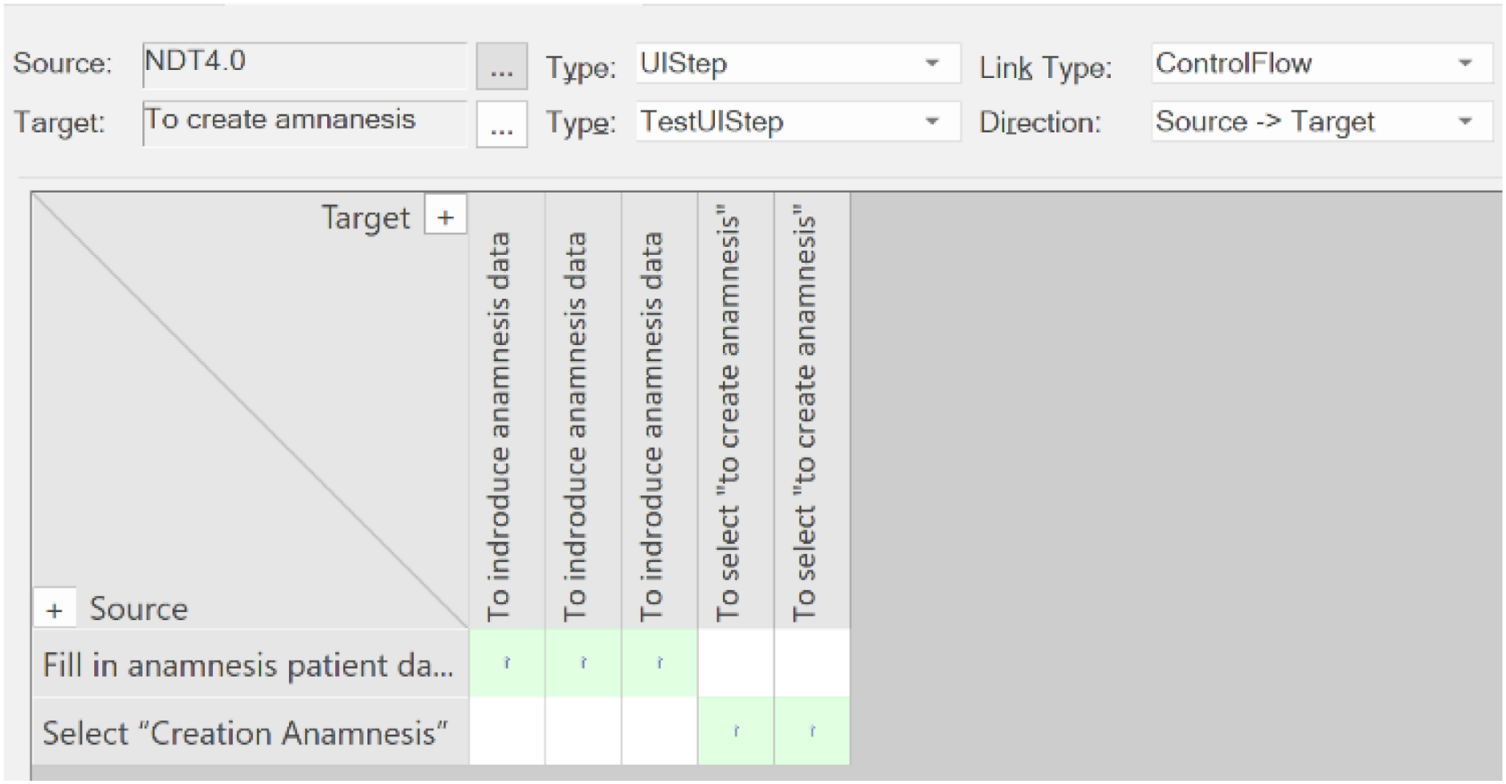
Traceability matrix for our example. This screen presents who the tool presents the traceability matrix that is automatically generated with our approach.

Secondly, the tool displays TraceLinks in a way not specifically oriented to each artifact, thus offering a global trace matrix that shows how all the sources are related to all the targets. The tool can be configured to select a set of sources and a set of targets. In this example, relationships between UIStep(s) and TestUIStep(s) were selected. The resulting matrix is shown in Fig. 12.

#### F. Automated monitoring of traces

**Figure 13:**
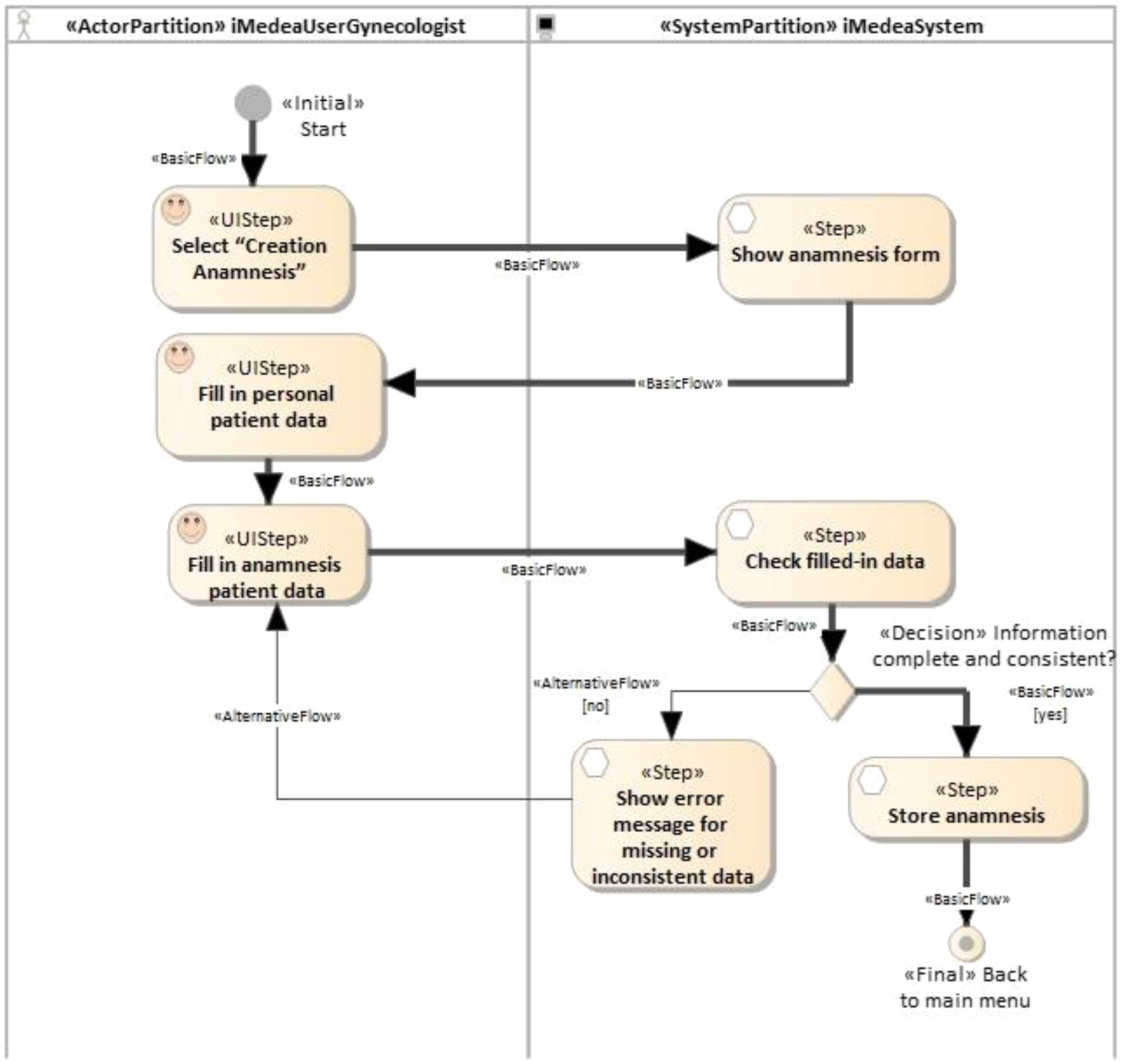
iMedea functional requirement: adding a new activity. This image presents an example to illustrate how the monitoring model helps in changing management.

Once TraceLink(s) have been created, the Quality plug-in checks consistency whenever a change is made—for instance, if a new activity like “Fill in personal patient data” is added to the activity diagram in Fig. 7. The updated activity diagram is shown in Fig. 13.

**Figure 14:**
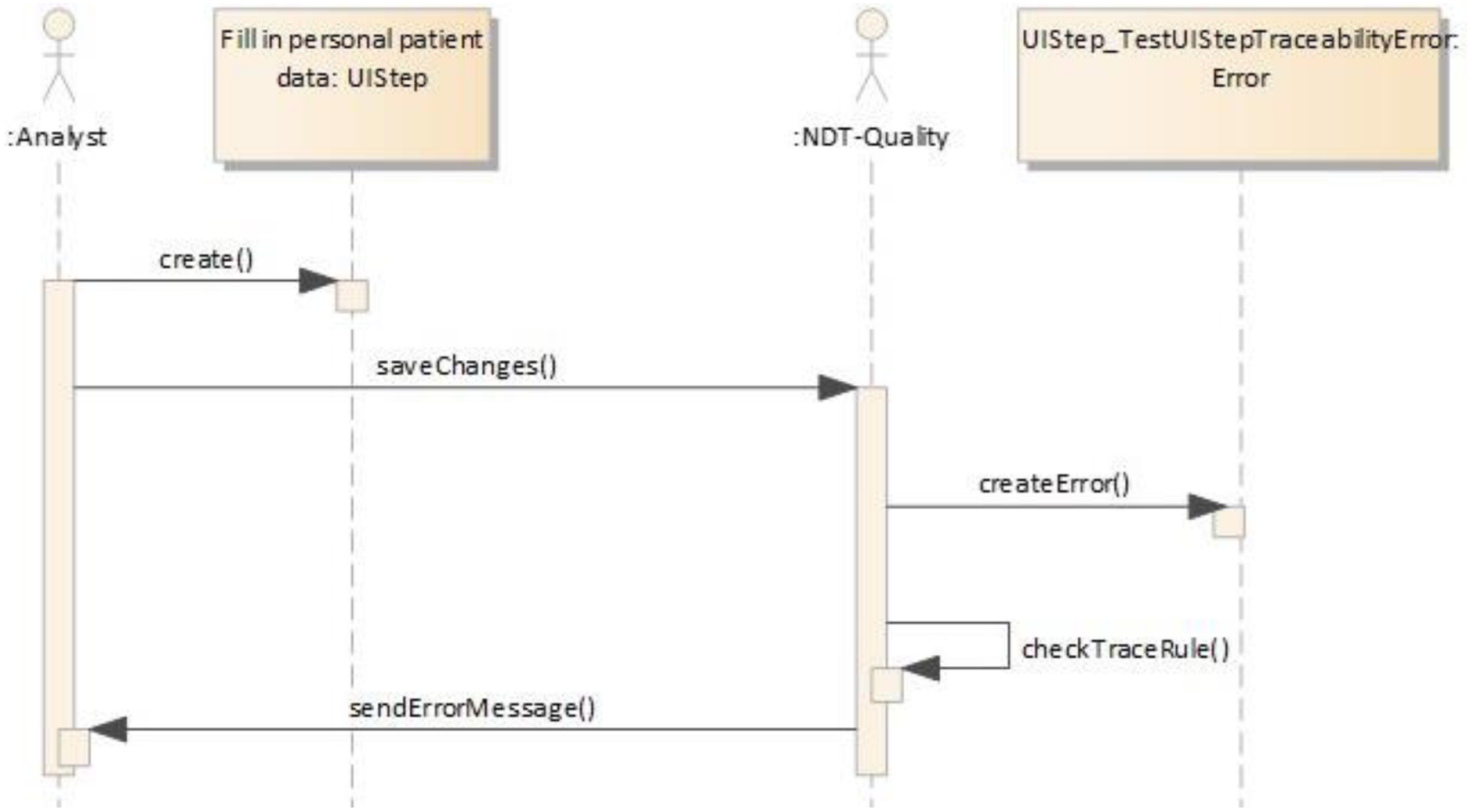
Monitoring process. The image presents a sequence model to illustrate the process of use of the monitoring model.

If the project is checked again or the project is closed, an error message will be displayed indicating that some information was lost for TestUIStep(s). The trace rule establishes that for each UIStep there has to be at least one TestUIStep. If this relationship is broken, an error is therefore caused. When this happens, the user could automatically navigate to a support panel that helps them to find and understand the error and to solve it. The quality control plug-in verifies that each Change is performed according to the TraceRule(s); it also generates Error(s) and Warning(s). If an inconsistency is detected, a message is sent to the developer with a recommendation to re-execute or update the transformations. The sequence diagram in Fig. 14 illustrates the process described above.

**Figure 15:**
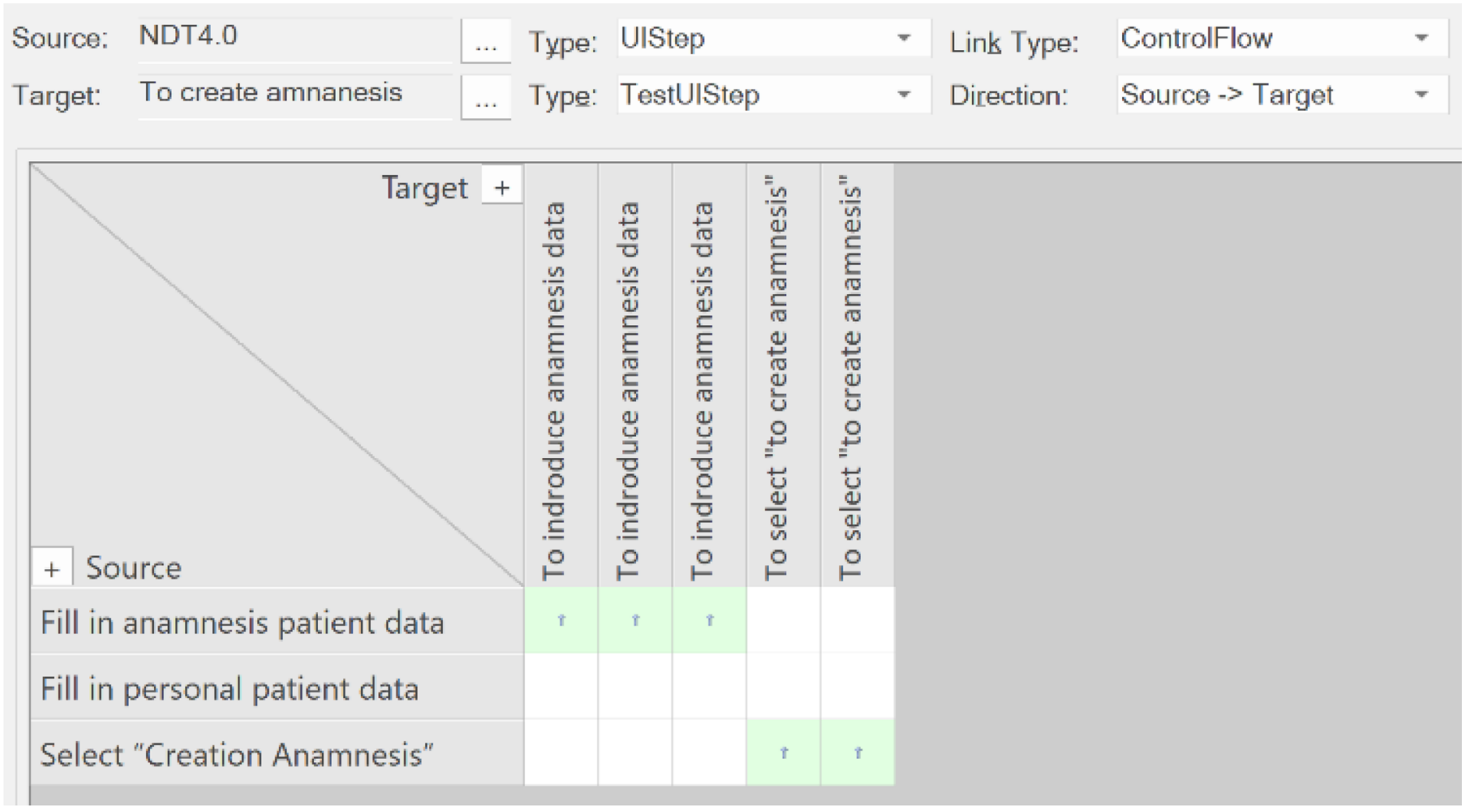
Traceability matrix for our example after change. This screen presents the traceability matrix generated after a changed.

In the traceability matrix shown in Fig. 15, the problem can clearly be seen: one of the UISteps is not connected to any TestUIStep(s).

In the matrix, the developer could click on “Fill in personal patient data” to navigate to this artifact and perform the appropriate act to keep the traceability consistent.

Alternatively, the developer could save the project with errors, which can be solved in future editions, to continue with the project. This would guarantee that all artifacts and models in the project are consistent.

The three tools presented above help to manage all aspects of the proposed traceability metamodel: creation of the ContextModel by the developer, generation of the TracingModel, analysis of changes following the MonitoringModel, and submission of error messages. They make (semi-)automatic traceability definition and management possible, and demonstrate that the proposed model-driven approach is capable of supporting those processes.

## Discussion

This article presents an MDE approach supporting automated, almost “for free”, trace management in the software construction process. The approach comprises a high-level, methodology-independent metamodel and its instantiation for a web methodology.

Traceability is very frequently referred to as a prerequisite to guarantee the quality of software products, but its actual implementation is usually complex and expensive, due to its requiring additional tools or a great amount of manual work.

The proposed approach makes it possible to create, maintain and manage traces as a by-product of model-driven development processes. Its integration in a particular MDE process requires the appropriate tool support, and has already been validated for the NDT methodology and tool suite. In this regard, it can be confirmed that MDE provides solutions for challenges **Ch.1. Automatic generation of traces** and **Ch.2. Tool support for trace model maintenance**, cited in “Materials and Methods”. This paper also demonstrates how the approach implements the automatic tracing of information; a task critical for information fusion.

The authors have used the proposed MDE-based traceability in several industrial projects, facilitating collaboration between stakeholders when addressing changes in requirements. Three projects worthy of mention are iMedea (G7 Innovation, 2021), DILECO (2021) and SAGE (2021, https://investigacion.us.es/sisius/sis_proyecto.php?idproy=29761).

iMedea (G7 Innovation, 2021) was briefly described in the *Materials and Methods* section of this paper. For numerical information about the impact of the proposed approach, it would be useful to look at traceability in this project, in which 78 use cases were defined. From the 390 UIStep(s) found in those use cases, 169 test cases were generated using the NDT tool suite’s Driver plug-in. These test cases comprised 1,178 TestUISteps. The proposed approach generated 1,178 TraceLink(s) from TestUISteps to UISteps. It is impossible to manage this number of TraceLink(s) manually. In addition to traces between use cases and test cases, traceability is also important for other artefacts like storage requirements, classes, and so on. The number of TraceLink(s) automatically generated in the iMedea project is currently close to one million. The importance of having a tool capable of managing traceability should therefore be clear.

The aim of the European project DILECO, carried out in collaboration with Airbus, was to develop and deploy Product Lifecycle Management (PLM) tools for A/C Ground Functional testing with Eco-design criteria to improve the sustainability of aircraft Final Assembly Lines (FAL) and the efficiency of the Ground System Test (GST) process end to end. This project offered an opportunity to assess the potential of the traceability matrix for managing heterogeneous, dispersed development teams in complex functional environments. The NDT tool was used in the project to develop a functional module for defining a control panel involving parameters for echo definition. The requirements specification of this module comprised 30 use cases and more than 200 activities. The development team was made up of about 15 people (this number varied during the project) and each result had to be validated with the general project team. Any change during product definition or validation was therefore critical and a great effort was needed to manage the specific aspects affected by each change in the overall system. The traceability matrix provided great support for easily finding connections between artifacts and for evaluating their impact on the rest of the project. The matrix is still used whenever system maintenance is performed.

Traceability was also a key factor in the SAGE project. The objective of this project was to design and develop a technological platform for the management of charging infrastructures for electric vehicles, buses, and trucks, under the electro mobility paradigm of a Smart-City ecosystem. This ecosystem is based on an open platform compatible with FIWARE (2021, https://www.fiware.org) and offers a comprehensive service to suppliers, maintainers, users, and those responsible for the city’s management. In this case, the whole team used the NDT tool to define the full structure of the system. A project team of about 15 people worked on the definition, design, implementation, and validation of the system. In this project, the set of requirements comprised 31 use cases describing more than 90 specific associated functional activities. Traceability was useful not only to coordinate changes during development but also for maintenance. After the first version of the product, the company needed to change part of the system. The traceability matrix made it possible to evaluate the cost of the change.

These experiences, and in particular the iMedea example described in the *Materials and Methods* section, demonstrate how effectively the proposed approach addresses challenge **Ch.4. Demonstration of the benefits of traceability in real examples managed by real users**, cited in “Materials and Methods”. For the authors of this paper, however, this is not enough. They plan to carry out a software experiment (López et al., 2020) based on the principles presented in Vegas (2017) to guarantee the benefits of the proposed approach in a more scientific manner. The industrial application of the approach would allow experiments to be organized within industry, offering an opportunity for further validation. The idea is to conduct controlled experiments in software companies to measure the value-added of this traceability approach for software development teams. As NDT is being used in several companies, we are confident of their experience and collaboration.

The proposed approach is also applicable and implementable for any other MDE methodology, in particular Web development approaches like UML-based Web Engineering (UWE) (Koch & Kozuruba, 2012), MockupDD (Rivero & Rossi, 2013), and Web Modeling Language (WebML) (Brambilla & Fraternali, 2014) One limitation for its extension is the existence of metamodels, transformations and tool support for the creation and management of traces.

## Conclusions

This article presents MDE as a mechanism for automating traceability in software engineering. Its main contributions are the following:

1. A generic approach to MDE traceability that explicitly includes a tracing mechanism.
2. An extension of a methodology by trace and change management.
3. An example of its application in a real context.

However, there are some problems and obstacles that will continue to limit the use of traceability approaches and delay the adoption of research prototypes in industry. One such problem is a lack of appropriate tool support. Another is that companies need to be persuaded of the benefits of traceability in their day-to-day software development business and the advantages it offers for improving the quality of their products. They need to understand the ROI of traceability and how to obtain the best results with minimum effort. Success stories like those provided in this work will help to disseminate and integrate traceability in the software development process. However, the authors believe it necessary to implement automatic ROI measurement in the tool as the best means of convincing companies. It is therefore necessary to continue working to improve the results in order to meet challenge 3 (**Ch.3. Return of investment (ROI) measurement**) cited in “Materials and Methods”.

One future task would be the implementation of a plug-in for an open source tool to allow further validation of the metamodel’s instantiation. The authors also plan to evaluate the approach using the benchmark proposed by Charrada et al. (2011) and to measure software quality improvements in current projects. A systematic, rigorous evaluation of the automated approach presented and implemented in this work might be based on the metrics proposed by Cleland-Huang (2006).

Another future work is to decouple the rules from the tool code in such a way that the TraceRules can be defined and interpreted by NDT “on the fly”, without the need to modify the code.

## Supplemental Information

10.7717/peerj-cs.817/supp-1 Supplemental Information 1 Code of Figure 6.

## Additional Information and Declarations

### Data Availability

The following information was supplied regarding data availability: The code is available in a Supplemental File.
